# Postpartum depression and associated risk factors during the COVID-19 pandemic

**DOI:** 10.1186/s13104-022-05991-8

**Published:** 2022-03-14

**Authors:** Clayton J. Shuman, Alex F. Peahl, Neha Pareddy, Mikayla E. Morgan, Jolyna Chiangong, Philip T. Veliz, Vanessa K. Dalton

**Affiliations:** 1 Department of Systems, Populations and Leadership, University of Michigan School of Nursing, 400 N. Ingalls, Ste. 4162, Ann Arbor, MI 48109-5842 USA; 2 Department of Obstetrics and Gynecology, University of Michigan Medical School, Ann Arbor, USA; 3 Applied Biostatistics Laboratory, University of Michigan School of Nursing, Ann Arbor, USA

**Keywords:** Postpartum depression, COVID-19, Maternal psychopathology, Breastfeeding

## Abstract

**Objective:**

To describe postpartum depression and associated risk factors among postpartum patients in the United States (US) between February and July 2020. This study used a cross-sectional descriptive design to collect survey data from a convenience sample of postpartum patients who lived in the US and delivered a live infant after the US declared COVID-19 a public health emergency.

**Results:**

Our sample included 670 postpartum patients who completed an online survey inclusive of the Edinburgh Postnatal Depression Scale (EPDS) and selected demographic items (e.g. NICU admission status, infant gestational age, infant feeding method). In our sample, 1 in 3 participants screened positive for postpartum depression and 1 in 5 had major depressive symptoms. Participants who fed their infants formula had 92% greater odds of screening positive for postpartum depression and were 73% more likely to screen positive for major depressive symptoms compared to those who breastfed or bottle-fed with their own human milk. Participants with infants admitted to a NICU had 74% greater odds of screening positive. Each 1 week increase in weeks postpartum increased the odds of screening positive by 4%. Participants who worried about themselves and their infants contracting COVID-19 had 71% greater odds of screening positive.

**Supplementary Information:**

The online version contains supplementary material available at 10.1186/s13104-022-05991-8.

## Introduction

Patients who delivered an infant during the coronavirus (COVID-19) pandemic have reported higher levels of stress during childbirth [1]. Additionally, 29.6% of pregnant patients assessed during the pandemic experience depressive symptoms [2]. Subsequently, COVID-19 has had a significant effect on peripartum mental health outcomes. COVID-19 continues to threaten public health, as infection rates in the United States increase dramatically and new variants emerge [3]. Accordingly, many cities, counties, and states within the United States have implemented or upheld mandates for the public to wear face masks to prevent the spread of COVID-19 [4]. Pregnant and postpartum patients are considered an at-risk population for severe COVID-19 infection and symptoms [5]. The emotional, social, and physical consequences associated with increased anxiety and confusion surrounding infection risk can adversely affect maternal mental health [6–8], placing postpartum patients at greater risk for postpartum depression [7–9]. Emerging research has begun to describe how the stressors associated with COVID-19 affect postpartum mental health [9–13]; however, few studies have been conducted in the United States which describe factors associated with postpartum depression during the COVID-19 pandemic.

As the COVID-19 pandemic and the public health response to it evolves, it is critical for clinicians and researchers to understand postpartum depression within the context of a global pandemic. Peripartum patients during the pandemic may have different experiences than those who delivered prior to the pandemic. Consequently, describing and understanding the risk factors for postpartum depression during this unprecedented time can inform clinical practice and policy. Accordingly, our report describes postpartum depression and associated risk factors of postpartum patients in the United Sates between February and July 2020.

## Main text

### Methods

This report is associated with the COVID-19 M.A.M.A.S. (Maternal Attachment, Mood, Ability, and Support) study which used a cross-sectional descriptive design to collect survey data from a convenience sample of postpartum patients who were at least 18 years of age**,** lived in the United States, and delivered a live infant after the United States declared COVID-19 a public health emergency (January 31, 2020) [14].

The study collected data on maternal mental health outcomes and related demographics and received ethics approval and a waiver of documentation of informed consent from the University of Michigan Institutional Review Board. All participants were provided with study information and informed that completing the survey signified their consent. Participants were recruited through group and individual accounts targeting peripartum women on major social media outlets. The web-based survey was developed specifically for this study, was available June 4-July 8, 2020, and included items to assess maternal mental health, self-efficacy, social support, and breastfeeding (see Additional file 1). This analysis describes results of the Edinburgh Postnatal Depression Scale (EPDS) [15] and associations among positive screens with potential demographic factors using logistic regression. Significance level was set at α = 0.05 for all analyses.

The EPDS is a valid and reliable screening tool that is widely used to identify depression symptoms in postpartum patients (in this study, Cronbach’s α = 0.88). Participants rate their agreement on 10 items using a 0–3 Likert scale. Total scores are calculated by summing scores across all items. A score of 10 or greater is considered a positive screen for postpartum depression and suggests further follow up is needed. A score of 13 or greater suggests presence of major depressive symptoms. Regardless of score, a positive response to the item about self-harm ideation results in a positive screen at both cut-off points [10, 13].

Explanatory factors included in the analyses were selected from available data in the survey demographic item section, including infant feeding (breastfeeding and/or pumping versus formula feeding), infant admission to a neonatal intensive care unit (NICU), gestational age of infant at birth, maternal age, number of weeks postpartum at time of survey completion, and three COVID-19 related items developed for this study. The COVID-19 items measured peripartum self-quarantining behaviors, worry of becoming infected with COVID-19 for self-and/or infant, and COVID-19 exposure (confirmed or suspected infection of self or family member).

## Results and discussion

The sample of those who completed the EPDS and all explanatory factors included 670 postpartum patients residing in 46 States. The sample was predominantly white and married (Table 1). Of participants, 38% (n = 256) screened positive for postpartum depression (score ≥ 10 or affirmative response to self-harm item) and, of these, 57% (n = 146; 21.8% of total sample) screened positive for major depressive symptoms (score ≥ 13 or affirmative response to self-harm item). Fifty-one participants (7.6% of total sample) provided an affirmative response to the self-harm item with most also scoring ≥ 13 (score < 10 = 5.9%; ≥ 10 and < 13 = 19.6%; ≥ 13 = 74.5%).

**Table 1:** Sample characteristics (n = 670)

	*M* (*SD*)	n (%)
Age	31.84 (3.88)	
Race white		595 (88.8)
Married		633 (94.5)
Weeks postpartum	7.46 (4.01)	
Infant gestational age at birth (in weeks)	38.84 (1.88)	
Breastfeeding or own human milk feeding		570 (85.1)
Infant admitted to a NICU		68 (10.1)
Worried about contracting COVID-19		
Baby only		51 (7.6)
Self only		42 (6.3)
Baby and self		458 (68.4)
Not worried at all		119 (17.8)
Self-quarantining perinatally (prenatal and/or postpartum)		569 (84.9)
Exposure to COVID-19 (confirmed self and/or household member and/or suspected self)		16 (2.4)
Positive screen for postpartum depression		256 (38)
Positive screen for major depressive symptoms		146 (21.8)

A positive screen for postpartum anxiety and depression = EPDS score of 10 or greater or a positive response to the item “The thought of harming myself has occurred to me.” Positive screen for major depressive symptoms = EPDS score of 13 or more or a positive response to the item “The thought of harming myself has occurred to me.”

Formula feeding, neonatal intensive care unit (NICU) admission, weeks postpartum, and worry of COVID-19 infection were associated with EPDS scores ≥ 10 (p < 0.05; Table 2). Only formula feeding was associated with EPDS scores ≥ 13. Participants who fed their infants formula had 92% greater odds of screening positive and were 73% greater odds to screen positive for major depressive symptoms compared to those who breastfed or bottle-fed with their own human milk. Participants who had infants admitted to a NICU had 74% greater odds of screening positive. Each 1 week increase in weeks postpartum increased a participant’s odds of screening positive by 4%. Participants who worried about themselves and their infants contracting COVID-19 had 71% greater odds of screening positive.

**Table 2:** Factors associated with EPDS positive screens for postpartum anxiety and depression (scores ≥ 10) and major depressive symptoms (scores ≥ 13) (n = 670)

Risk factor	≥ 10 EPDS score	≥ 13 EPDS score
	OR (95% CI)	OR (95% CI)
Formula feeding	1.92** (1.23–3.00)	1.73* (1.06–2.79)
Age	0.96 (0.9–1.00)	0.98 (0.93–1.03)
NICU admission	1.74* (1.03–2.93)	1.26 (0.69–2.24)
Weeks postpartum	1.04* (1.00–1.09)	1.04 (0.99–1.09)
Race not white	1.20 (0.72–1.99)	0.97 (0.52–1.74)
Not married	0.98 (0.48–1.96)	1.93 (0.92–3.91)
COVID-19 infection worry for self and baby	1.71** (1.20–2.45)	1.46 (0.96–2.25)
Self-quarantine during pregnancy and/or postpartum	1.17 (0.74–1.86)	1.04 (0.62–1.81)
Exposure to COVID-19 (positive test self, member of household, or suspected self)	1.18 (0.40–3.30)	1.56 (0.48–4.46)

The dependent in this analysis is a positive screen for postpartum anxiety and depression using the EPDS coded so that 0 = an EPDS score of 0–9 and 1 = an EPDS score of 10 or greater or a positive response to the item “The thought of harming myself has occurred to me.” Positive screen for major depressive symptoms was coded as 1 = EPDS score of 13 or more or a positive response to the item “The thought of harming myself has occurred to me.”

* = p < .05, ** = p < .01

Within this sample of postpartum patients recruited during the early stages of the COVID-19 pandemic in the US, over 1 in 3 participants (38.2%) screened positive for postpartum depression (EPDS score ≥ 10) and 1 in 5 (21.8%) screened positive for major depressive symptoms (EPDS score ≥ 13). The rate of postpartum depression in this convenience sample is notably higher than those reported in the US pre-pandemic (6.5–12.9%) [8]. The relatively high rate of postpartum depression identified in this study may be associated with pandemic-related events that resulted in significant changes to patients’ lives and peripartum experiences. Pandemic-related events may include the emotional and economic impact of social distancing, cessation of routine childbirth visitation policies, online breastfeeding and parenting classes, transitions to telemedicine, and COVID-19 infection worry [10].

Public mandates for social distancing, mask-wearing, and stay-at-home orders, may have led to decreased social support important to breastfeeding and COVID-related worry. Snyder and colleagues found patients who delivered an infant during the pandemic had significantly lower levels of social support during the postpartum period [16]. Additionally, Brown and colleagues found that 27% of women struggled to get adequate breastfeeding support during COVID-19 [17]. Patients who lack the necessary support to breastfeed are less likely to breastfeed and more likely to formula feed [18]. Further, patients who experience postpartum depression are less likely to breastfeed [19]. Decreased social support during COVID-19 may help to explain why formula feeding contributed to greater odds of screening positive for postpartum depression in our sample. Clinics and healthcare systems are encouraged to bolster their breastfeeding support and resources, with an emphasis on social support, to help patients achieve their breastfeeding goals and reduce their risk for postpartum depression.

A study conducted by Vance and colleagues during the same time period as our study found that NICU admission during COVID-19 resulted in significant strain on family well-being and finances [20]. Additionally, visitation restrictions in NICUs caused significant distress for postpartum patients [21–23]. Postpartum patients of NICU infants may have had higher odds of screening positive for postpartum depression due to the strain of NICU admission on family well-being and due to restrictive visitation policies.

We also found that each 1 week increase in weeks postpartum increased a participant’s odds of screening positive for postpartum depression. This finding aligns with the psychopathology of postpartum depression that starts within a few weeks of giving birth and can worsen over time. It is possible that identification and treatment of postpartum depression was stalled during the COVID-19 pandemic due to reduced access to providers, changes in routine peripartum care visits, and limited telehealth options early in the pandemic.

Further, we found those who worried about themselves or their infants contracting COVID-19 had greater odds of screening positive for postpartum depression. Heightened social stress correlates to higher incidences of depression [24]. Accordingly, in our sample of participants, increased social stress (e.g. fear and worry about COVID-19 infection) may have contributed to increased odds of screening positive for postpartum depression. Clinicians working with peripartum patients during the COVID-19 pandemic are encouraged to implement strategies that decrease peripartum worry related to COVID-19, such as discussing COVID-19 vaccination and offering peer support.

In our sample, 7.6% of participants reported having thoughts about harming themselves. Among those who screened positive for postpartum depression or major depressive symptoms, 18.75% reported having self-harm thoughts. This is particularly concerning given that even before the COVID-19 pandemic, suicidality and suicide rates were rising among pregnant and recently pregnant women [25]. Thus, it is critical to understand the contextual factors (like those created by the COVID-19 pandemic) that may increase suicide risk so that healthcare systems and providers can be better prepared for future events that may similarly disrupt care, create isolation, or exacerbate existing depression and anxiety.

## Conclusion

Postpartum mental health is an important but relatively overlooked outcome in the COVID-19 pandemic. Pandemic sequelae such as face mask mandates, fear of infection, NICU visitor restrictions, and social isolation may have helped to slow infection rates, but they may also have contributed to worse postpartum mental outcomes. More research is needed to understand how and why pandemic sequalae are associated with peripartum mental health outcomes. Health systems must ensure adequate infrastructure to support the diverse needs of peripartum patients, including educational (e.g. childbirth, breastfeeding, parenting) and psychosocial (e.g. screening and management of mental health conditions and social determinants of health) services during distracting situations like pandemics, especially as COVID-19 continues to threaten public health.

## Limitations

Although this study was one of the first to describe risk factors for postpartum depression in the United States during the early months of the COVID-19 pandemic, there are limitations to consider. Our study used a convenience sample of patients recruited only through social media and may not be representative of the entire postpartum population during this time. Despite efforts to recruit a more diverse sample, our sample was mostly white and married. While we observed representation across states, it is important to consider how varying state and regional infection spikes may have affected participant responses. Further, this study did not collect data on all known contributors to postpartum depression nor was it designed to comprehensively identify all new risk factors associated with the COVID-19 pandemic. Despite these limitations, our findings inform research, practice, and policy.

## Supplementary Information

**Additional file 1: **COVID-19 M.A.M.A.S. (Maternal Attachment, Mood, Ability, and Support) Full Survey.

## Data Availability

The datasets used and/or analyzed during the current study are available from the corresponding author on reasonable request.

## Abbreviations

COVID-19: Coronavirus disease 2019/COVID-19 pandemic
US: United States
EPDS: Edinburgh Postnatal Depression Scale
NICU: Neonatal intensive care unit
