# Supplementary material for: Postpartum depression and associated risk factors during the COVID-19 pandemic
Source: BMC Res Notes. 2022 Mar 14;15:102. doi: 10.1186/s13104-022-05991-8 (PMC8919141; doi:10.1186/s13104-022-05991-8)
Supplement: Supplementary file 1 — Additional file 1: COVID-19 M.A.M.A.S. (Maternal Attachment, Mood, Ability, and Support) Full Survey. [file 13104_2022_5991_MOESM1_ESM.docx]

**Additional file 1: COVID-19 M.A.M.A.S. (Maternal Attachment, Mood, Ability, and Support) Full Survey**

**PI: Clayton J. Shuman, PhD, MSN, RN; Assistant Professor, University of Michigan School of Nursing; clayshu@umich.edu**

Edinburgh Postnatal Depression Scale

For the following questions please select that answer that comes closest to how you have felt in the past 7 days, not just how you feel today. In the past 7 days…

1. I have been able to laugh and see the funny side of things.
   1. As much as I always could
   2. Not quite so much now
   3. Definitely not so much now
   4. Not at all
2. I have looked forward with enjoyment to things.
   1. As much as I ever did
   2. Rather less than I used to
   3. Definitely less than I used to
   4. Hardly at all
3. I have blamed myself unnecessarily when things went wrong.
   1. Yes, most of the time
   2. Yes, some of the time
   3. Not very often
   4. No, never
4. I have been anxious or worried for no good reason.
   1. No, not at all
   2. Hardly ever
   3. Yes, sometimes
   4. Yes, very often
5. I have felt scared or panicky for no very good reason.
   1. Yes, quite a lot
   2. Yes, sometimes
   3. No, not much
   4. No, not at all
6. Things have been getting on top of me.
   1. Yes, most of the time I haven’t been able
      to cope at all.
   2. Yes, sometimes I haven’t been coping as well as usual.
   3. No, most of the time I have coped quite well.
   4. No, I have been coping as well as ever
7. I have been so unhappy that I have had difficulty sleeping.
   1. Yes, most of the time
   2. Yes, sometimes
   3. Not very often
   4. No, not at all
8. I have felt sad or miserable.
   1. Yes, most of the time
   2. Yes, quite often
   3. Not very often
   4. No, not at all
9. I have been so unhappy that I have been crying.
   1. Yes, most of the time
   2. Yes, quite often
   3. Only occasionally
   4. No, never
10. The thought of harming myself has occurred to me.
    1. Yes, quite often
    2. Sometimes
    3. Hardly ever
    4. Never

Birth Memories and Recall Questionnaire

The next set of questionnaire asks about what your memories of the birth of your most recent child are like, and how you feel when you remember the birth now. If you had a caesarean under general anesthetic, please answer the questions as best you can for your memory of the experience immediately before and after.

|  | Strongly Disagree |  |  |  |  |  | Strongly Agree |
| --- | --- | --- | --- | --- | --- | --- | --- |
| 1. My emotions at the time were extremely positive | 1 | 2 | 3 | 4 | 5 | 6 | 7 |
| 1. My emotions at the time were extremely negative | 1 | 2 | 3 | 4 | 5 | 6 | 7 |
| 1. I experienced mixed positive and negative emotions at the time | 1 | 2 | 3 | 4 | 5 | 6 | 7 |
| 1. While recalling the birth now, my emotions are extremely positive | 1 | 2 | 3 | 4 | 5 | 6 | 7 |
| 1. While recalling the birth now, I am experiencing mixed positive and negative emotions | 1 | 2 | 3 | 4 | 5 | 6 | 7 |
|  | Not At All |  |  |  |  |  | Very Much So |
| 1. While remembering the birth now, I relive visual impressions I had during the birth | 1 | 2 | 3 | 4 | 5 | 6 | 7 |
| 1. While remembering the birth now, I relive the bodily sensations I had during the birth | 1 | 2 | 3 | 4 | 5 | 6 | 7 |
| 1. While remembering the birth now, I feel as though I am reliving it and it is happening now, not in the past | 1 | 2 | 3 | 4 | 5 | 6 | 7 |
| 1. While remembering the birth now, I relive the sound(s) I heard during the birth | 1 | 2 | 3 | 4 | 5 | 6 | 7 |
|  | Strongly Disagree |  |  |  |  |  | Strongly Agree |
| 1. The experience of birth has coloured the way I think and feel about other experiences | 1 | 2 | 3 | 4 | 5 | 6 | 7 |
| 1. The experience of birth has become central to the way I understand myself and the world | 1 | 2 | 3 | 4 | 5 | 6 | 7 |
| 1. The experience of birth was a turning point in my life | 1 | 2 | 3 | 4 | 5 | 6 | 7 |
| 1. I often think about the effects the experience of birth will have on my future | 1 | 2 | 3 | 4 | 5 | 6 | 7 |
|  | None At All |  |  |  |  |  | A Lot |
| 1. As I recall the birth I can remember smells | 1 | 2 | 3 | 4 | 5 | 6 | 7 |
| 1. As I recall the birth, I can remember tastes | 1 | 2 | 3 | 4 | 5 | 6 | 7 |
| 1. As I recall the birth, I can remember sounds | 1 | 2 | 3 | 4 | 5 | 6 | 7 |
| 1. As I recall the birth, I can remember touch | 1 | 2 | 3 | 4 | 5 | 6 | 7 |
|  | Never |  |  |  |  |  | All The Time |
| 1. My memory for the birth (or parts of the memory) comes to me 'out of the blue' without me trying to think about it | 1 | 2 | 3 | 4 | 5 | 6 | 7 |
| 1. Things that happen now can unexpectedly bring up memories of the birth (or parts of memories) | 1 | 2 | 3 | 4 | 5 | 6 | 7 |
|  | Strongly Disagree |  |  |  |  |  | Strongly Agree |
| 1. My memory for the birth comes to me as a logical, coherent series of events with no major gaps | 1 | 2 | 3 | 4 | 5 | 6 | 7 |
| 1. My memory for the birth is fragmented, i.e. it comes in bits and pieces with bits missing | 1 | 2 | 3 | 4 | 5 | 6 | 7 |

Interpersonal Support Evaluation List-12

Following is a list of statements each of which may or may not be true about you. For each statement circle "definitely true" if you are sure it is true about you and "probably true" if you think it is true but are not absolutely certain. Similarly, you should circle "definitely false" if you are sure the statement is false and "probably false" if you think it is false but are not absolutely certain.

1. If I wanted to go on a trip for a day (for example, to the country or mountains), I would have a hard time finding someone to go with me.
   1. Definitely false
   2. Probably false
   3. Probably true
   4. Definitely true
2. I feel that there is no one I can share my most private worries and fears with.
   1. Definitely false
   2. Probably false
   3. Probably true
   4. Definitely true
3. If I were sick, I could easily find someone to help me with my daily chores.
   1. Definitely false
   2. Probably false
   3. Probably true
   4. Definitely true
4. There is someone I can turn to for advice about handling problems with my family.
   1. Definitely false
   2. Probably false
   3. Probably true
   4. Definitely true
5. If I decide one afternoon that I would like to go to a movie that evening, I could easily find someone to go with me.
   1. Definitely false
   2. Probably false
   3. Probably true
   4. Definitely true
6. When I need suggestions on how to deal with a personal problem, I know someone I can turn to.
   1. Definitely false
   2. Probably false
   3. Probably true
   4. Definitely true
7. I don't often get invited to do things with others.
   1. Definitely false
   2. Probably false
   3. Probably true
   4. Definitely true
8. If I had to go out of town for a few weeks, it would be difficult to find someone who would look after my house or apartment (the plants, pets, garden, etc.).
   1. Definitely false
   2. Probably false
   3. Probably true
   4. Definitely true
9. If I wanted to have lunch with someone, I could easily find someone to join me.
   1. Definitely false
   2. Probably false
   3. Probably true
   4. Definitely true
10. If I was stranded 10 miles from home, there is someone I could call who could come and get me.
    1. Definitely false
    2. Probably false
    3. Probably true
    4. Definitely true
11. If a family crisis arose, it would be difficult to find someone who could give me good advice about how to handle it.
    1. Definitely false
    2. Probably false
    3. Probably true
    4. Definitely true
12. If I needed some help in moving to a new house or apartment, I would have a hard time finding someone to help me.
    1. Definitely false
    2. Probably false
    3. Probably true
    4. Definitely true

Being a Mother Scale-13

For each of the next statements think about how you have been feeling over the past 2–3 weeks. There are no right or wrong answers.

1. I have felt confident about looking after my baby/toddler.
   1. Yes, most or all of the time
   2. Yes, some of the time
   3. No, not very often
   4. No, rarely or never
2. I have missed the life I had before I became pregnant with this baby/toddler (*or for adoptive mothers*: before I had this baby/toddler).
   1. No, rarely or never
   2. No, not very often
   3. Yes, some of the time
   4. Yes, most or all of the time
3. I have found it hard to cope when my baby/toddler cries.
   1. No, rarely or never
   2. No, not very often
   3. Yes, some of the time
   4. Yes, most or all of the time
4. I have felt close to my baby/toddler.
   1. Yes, most or all of the time
   2. Yes, some of the time
   3. No, not very often
   4. No, rarely or never
5. I have felt lonely or isolated.
   1. No, rarely or never
   2. No, not very often
   3. Yes, some of the time
   4. Yes, most or all of the time
6. I have felt bored.
   1. No, rarely or never
   2. No, not very often
   3. Yes, some of the time
   4. Yes, most or all of the time
7. I have felt unsupported.
   1. No, rarely or never
   2. No, not very often
   3. Yes, some of the time
   4. Yes, most or all of the time
8. I have felt alright about asking people for help or advice when I needed to.
   1. Yes, most or all of the time
   2. Yes, some of the time
   3. No, not very often
   4. No, rarely or never
9. I have felt nervous or uneasy around my baby/toddler.
   1. No, rarely or never
   2. No, not very often
   3. Yes, some of the time
   4. Yes, most or all of the time
10. I have been worried that something would happen to my baby/toddler.
    1. No, rarely or never
    2. No, not very often
    3. Yes, some of the time
    4. Yes, most or all of the time
11. I have been annoyed or irritated with my baby/toddler.
    1. No, rarely or never
    2. No, not very often
    3. Yes, some of the time
    4. Yes, most or all of the time
12. I worry I am not as good as other mothers.
    1. No, rarely or never
    2. No, not very often
    3. Yes, some of the time
    4. Yes, most or all of the time
13. I have felt guilty.
    1. No, rarely or never
    2. No, not very often
    3. Yes, some of the time
    4. Yes, most or all of the time
14. If you have found being a mother very stressful, very difficult, or unenjoyable, why do you think this is?

Complete the following statement.

1. Most of the time I feed my infant by:
   1. Breastfeeding
   2. Pumping my own milk and feeding it to my infant in a bottle or feeding tube
   3. Using human donor milk
   4. Formula feeding

Breastfeeding Experience Scale

Rate the following on a scale of 1-5 where 1 indicates not at all, and 5 indicates an unbearable amount.

**Not at all** **Unbearable**

1 2 3 4 5

1. Sore nipples.

1 2 3 4 5

1. Cracked nipple.

1 2 3 4 5

1. Breast engorgement.

1 2 3 4 5

1. Baby having difficulty in latching on.

1 2 3 4 5

1. Baby reluctant to nurse due to sleepiness.

1 2 3 4 5

1. Baby reluctant to nurse due to fussiness.

1 2 3 4 5

1. Breast infection.

1 2 3 4 5

1. Leaking breasts.

1 2 3 4 5

1. Baby nursing too frequently.

1 2 3 4 5

1. Worry of not having enough milk.

1 2 3 4 5

1. Baby having difficulty in sucking.

1 2 3 4 5

1. Feeling very tired.

1 2 3 4 5

1. Worry that baby was not getting enough milk.

1 2 3 4 5

1. Difficulty in positioning baby.

1 2 3 4 5

1. Worry about baby’s weight gain.

1 2 3 4 5

1. Feeling tense and overwhelmed.

1 2 3 4 5

1. Feeling embarrassed when nursing.

1 2 3 4 5

1. Difficulty in combining work and breastfeeding.

1 2 3 4 5

Maternal Infant Bonding Questionnaire

These next questions are about your feelings for your child in the first few weeks. Some adjectives are listed below which describe some of the feeling’s mothers have towards their baby in the **first** **weeks** after they were born. Please select the options which best describes how you felt in the **first few weeks**.

1. Loving
   1. Very much
   2. A lot
   3. A little
   4. Not at all
2. Resentful
   1. Very much
   2. A lot
   3. A little
   4. Not at all
3. Neutral or feeling nothing
   1. Very much
   2. A lot
   3. A little
   4. Not at all
4. Joyful
   1. Very much
   2. A lot
   3. A little
   4. Not at all
5. Dislike
   1. Very much
   2. A lot
   3. A little
   4. Not at all
6. Protective
   1. Very much
   2. A lot
   3. A little
   4. Not at all
7. Disappointed
   1. Very much
   2. A lot
   3. A little
   4. Not at all
8. Aggressive
   1. Very much
   2. A lot
   3. A little
   4. Not at all

Please complete the following.

1. Age:
2. Ethnicity
   1. Asian
   2. Black
   3. Caucasian
   4. Hispanic/Latinx
   5. Native American
   6. Pacific Islander
   7. Prefer not to answer
   8. Other:
3. Select the following that apply to you at the time of delivery in regard to COVID-19.
   1. I tested positive
   2. Someone in my home tested positive
   3. I know I had COVID-19 but did not get tested
   4. I self-quarantined during my pregnancy
   5. I self-quarantined after I gave birth
   6. Explain:
4. Gestational age of my baby when they were born.
   1. 24, 25, 26, 27, 28
5. My baby was admitted to the NICU when they were born.
   1. Yes
   2. No
6. If there is any other information that you would like to provide, please do so here:

If you are thinking about suicide, hurting yourself or your baby, please call 911 right away.

If you are concerned about your mental wellbeing, or are concerned about a loved one, please take a look at these resources:

- [National Suicide Prevention Lifeline](https://suicidepreventionlifeline.org/talk-to-someone-now/), available 24/7: 1-800-273-8255
- [Substance Abuse and Mental Health National Helpline](https://www.samhsa.gov/find-help/national-helpline), available 24/7: 1-800-662-4357
- [Postpartum Support International](http://www.postpartum.net/), not for emergencies, COVID-19 information: 800-944-4PPD (4773)
- [Depression during and after pregnancy: A resource for women, their families and friends](https://mchb.hrsa.gov/sites/default/files/mchb/MaternalChildHealthTopics/maternal-womens-health/depression_during_and_after_pregnancy_en.pdf) from the U.S. Department of Health and Human Services, Health Resources and Services Administration, Office of Maternal and Child Health
